# Massive invasion on a Triatominae insectarium (Hemiptera: Heteroptera: Reduviidae) by *Attagenus fasciatus* (Thunberg, 1795) (Coleoptera: Dermestidae: Attageninae)

**DOI:** 10.1590/0037-8682-0150-2023

**Published:** 2023-07-24

**Authors:** Jader de Oliveira, Jiří Háva, João Aristeu da Rosa, Hélcio Reinaldo Gil-Santana

**Affiliations:** 1 Universidade de São Paulo, Faculdade de Saúde Pública, Laboratório de Entomologia em Saúde Pública, São Paulo, SP, Brasil. Universidade de São Paulo Faculdade de Saúde Pública Laboratório de Entomologia em Saúde Pública São Paulo SP Brasil; 2 Private Entomological Laboratory and Collection, Rýznerova 37/37, CZ-252 62 Únětice u Prahy, Prague-west, Czech Republic. Private Entomological Laboratory and Collection Rýznerova 37/37 CZ-252 62 Únětice u Prahy Prague-west Czech Republic; 3 Universidade Estadual Paulista, Faculdade de Ciências Farmacêuticas, Araraquara, SP, Brasil. Universidade Estadual Paulista Faculdade de Ciências Farmacêuticas Araraquara SP Brasil; 4 Instituto Oswaldo Cruz, Laboratório de Diptera, Rio de Janeiro, RJ, Brasil. Instituto Oswaldo Cruz Laboratório de Diptera Rio de Janeiro RJ Brasil

**Keywords:** Living collection, Triatominae insectarium, Biological collections, Conservation, Pests

## Abstract

**Background:**

Triatominae are vectors of the protozoan *Trypanosoma cruzi*, the causative agent of Chagas disease. This study aims to report an infestation on Triatominae colonies by a beetle, previously identified as a pest.

**Methods:**

The management of these colonies should be improved to maximize their usefulness, and factors that may cause harm to them should be avoided as much as possible.

**Results:**

This is the first report on a coleopteran infestation on living Triatominae colonies worldwide.

**Conclusions:**

The present record provides an important warning to researchers who maintain insectaries in general, especially those who rear triatomines, to carry protective measures against such invasions.

The subfamily Triatominae currently includes approximately 160 extant species defined by their blood-sucking habit^1^^,^^2^. All are considered potential vectors of the protozoan *Trypanosoma cruzi*, the causative agent of Chagas disease or American trypanosomiasis, which remains a serious health problem in most Latin American countries^3^. 

Therefore, several species are reared intensively in many laboratories. *Rhodnius prolixus*, for example, is one of the most important vectors of Chagas disease, and over the past century, intense investigations have been carried on it as an insect model to explore important aspects of metabolism, endocrinology, and physiology as it has been proven to be a highly adaptable laboratory insect^4^^,^^5^. 

Despite all the intensive and extensive rearing of triatomines in insectaries, information on plagues attacking these artificial colonies are lacking; we only found a paper^6^ published in an obscure Brazilian Journal recording insects which might damage rearing triatomine insectaries. 

It recorded a single massive attack by ants (Hymenoptera: Formicidae), possibly belonging to the genus *Eciton* Latreille, 1804, on a Triatominae insectary in São Paulo in 1954. Additionally, Corrêa et al^6^ recorded the following insects as plagues of triatomine insectaries: 1) cockroaches (Blattodea); the most common were *Blatella germanica* (Linnaeus, 1758) and *Periplaneta americana* (Linnaeus, 1758); 2) flies of the family Chloropidae, which invaded insectaries in high numbers; and 3) *Telenomus fariai* Costa Lima, 1927 (Hymenoptera: Scelionidae), a parasitoid of triatomine eggs. 

Coscarón et al^7^ compiled information on the predators and parasitoids of Triatominae. In this paper, they reported three topics: 1) an updated list of the parasitoids and predators that attack Triatominae; 2) statements about the relationship between the predators and parasitoids and their hosts; and 3) a discussion about the more important species that can be used for biological control. Moreover, they provided tables with a bibliography of the biology and systematics of approximately 120 species of Triatominae predators and parasitoids. This study documented, for the first time, an infestation of Triatominae colonies by a beetle, previously identified as a pest.

The Triatominae colonies were maintained in crystallizers (a height of 25 cm x a diameter of 40 cm) using a filter paper bottom and pressed Kraft paper support to increase the contact surface and were closed with two woven cloths held together with the aid of a rubber band. Currently, 150 colonies of 50 different species are maintained in the Triatominae insectary. During a maintenance cleaning of the colonies, larvae and adults of a coleopteran species were observed inside colonies of *R. prolixus* and *R. neglectus* (Figure 1A, 1B, 1C, 1D, and 1E ). The colonies were immediately transferred to an isolation room to avoid contamination with other colonies. 

All photographs were captured by the first author (JO) using the digital camera of a smartphone (Iphone XR) for the alive specimens in the lab (Figure 1A, 1B, 1C, 1D and 1E ) and a digital camera Leica DMC 2905 attached to a Leica M205C stereomicroscope for the fixed adults (Figure 2A, 2B ). 

A high number of individuals both in the larval stage (> 50 specimens) (Figure 1B and 1C ) and adults (> 10 specimens) (Figure1 D and 1E ) of *Attagenus fasciatus* (Thunberg, 1795) (Coleoptera: Dermestidae: Attageninae) in the colonies of *R. prolixus* and *R. neglectus* (Triatominae) was unexpectedly recorded for the first time. The coleopteran species was identified based on morphological characteristics^8^ and was confirmed by Dr. Jiří Háva through analyzing the high-resolution images of the adults (Figure 2A and 2B ).

**FIGURE 1A-E:**
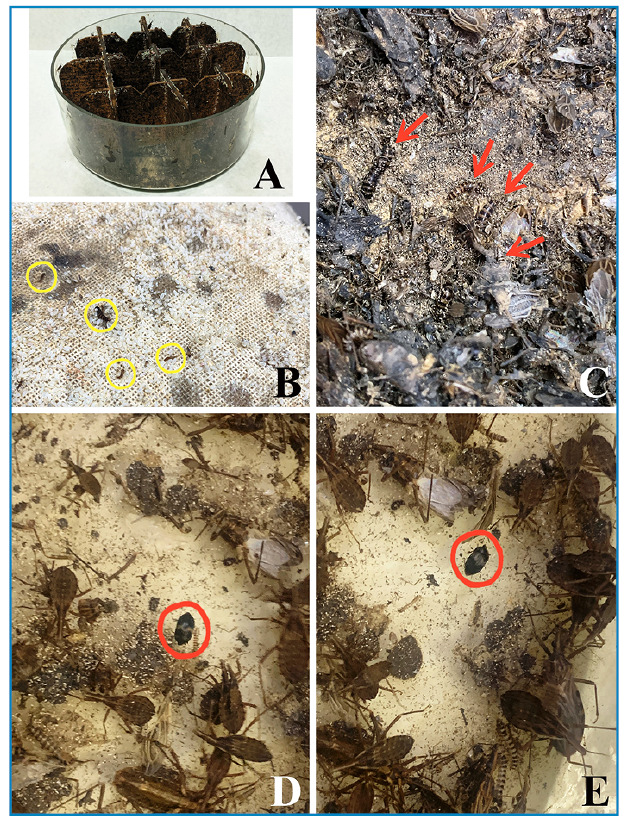
Specimens of *Attagenus fasciatus* photographed inside *Rhodnius* colonies. A: crystallizer for the rearing of the Triatominae colony; B and C larvae; D and E adults. Yellow circles: larvae amid egg shells; red arrows: larvae in the middle of the colony substrate; red circles: adults in the middle of the colony.

**FIGURE 2A-B:**
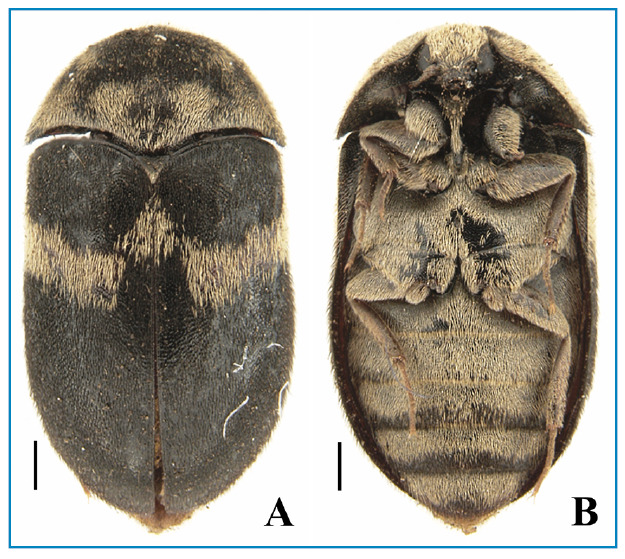
Adult specimens of *Attagenus fasciatus*; A: dorsal view and B: ventral view Scale bars: 0.5 mm.

*Attagenus fasciatus* is a cosmopolitan species^9^^,^^10^, whose larvae can damage woolen clothes, carpets, blankets, feathers, hair, and preserved museum specimens, whereas adults feed solely on pollen and nectar^11^^,^^12^. Several studies on the biology of immature and adult *A. fasciatus* have been conducted by Ali^12^. 

All colonies were checked to ensure that contamination had dissipated, and only two colonies showed contamination. The latter were cleaned and kept for 30 days in an isolation drying oven at 35 degrees after removing the live triatomines to ensure that no specimens remained there. For cleaning, the crystallizer, paper background, internal separator, cloth, and rubber cloth were completely replaced. The larvae and adults of *Attagenus fasciatus* were fixed in 100% ethanol and deposited in the museum collection as pests of Triatominae insectaries.

The diversity of the Dermestidae associated with insects demonstrates their remarkable ecological and trophic adaptability. Their larvae are opportunistic scavengers that are capable of exploiting different food substrates, including inhabiting bird nests as reported by Turienzo & Iorio^13^, similar to the environment where we found certain species of Triatominae^13^^,^^14^. This association between Dermestidae and bird nests underscores the significance of the current research and emphasizes the need to further understand the interactions between these insects and Triatominae vectors in a shared environment.

Finally, a more detailed analysis of the impact of pests on colonies revealed some important findings. Coleopteran subjects have a significant proliferative capacity, resulting in infestations that can negatively affect the environment. Both immature forms and adults feed on dead insects and colony waste. Although an immediate observable damage could not be identified, further investigations are required to accurately assess the effects of these insects on colonies. Additionally, it is important to consider the potential competition for space with triatomines, which might lead them to feed on fertile eggs, causing harm to the colony or resulting in other adverse effects.

These aspects should be addressed in subsequent studies to provide a more comprehensive understanding of the effects of this pest. This is the first report on a coleopteran infestation on living Triatominae colonies worldwide. This record is an important warning to researchers who maintain insectaries in general, especially those who rear triatomines, to carry protective measures against such invasions on their insectaries.
